# Neoadjuvant PD-1 inhibitor combined with FLOT versus SOX for locally advanced gastric cancer: a retrospective cohort study

**DOI:** 10.3389/fimmu.2026.1782029

**Published:** 2026-02-25

**Authors:** Zhenshun Li, Xin Zhang, Lili Duan, Wanli Yang, Liu Hong

**Affiliations:** 1 Department of Digestive Surgery, Xijing Hospital of Digestive Diseases, Fourth Military Medical University, Xi’an, Shaanxi, China; 2 State Key Laboratory of Holistic Integrative Management of Gastrointestinal Cancers and National Clinical Research Center for Digestive Diseases, Xijing Hospital of Digestive Diseases, Fourth Military Medical University, Xi’an, Shaanxi, China; 3 Department of Radiology, Second Affiliated Hospital of Naval Medical University, Shanghai, China; 4 Department of Radiology, 920th Hospital of Joint Logistics Support Force, PLA, Kunming, China

**Keywords:** gastric cancer, neoadjuvant chemotherapy, pathological response, PD-1 inhibitor, survival

## Abstract

**Background:**

Neoadjuvant immunochemotherapy is increasingly used for resectable locally advanced gastric cancer (LAGC) and gastroesophageal junction (EGJ) adenocarcinoma, yet the optimal chemotherapy backbone for PD-1 blockade remains unclear. We compared neoadjuvant PD-1 inhibitor plus S-1+oxaliplatin (SOX) versus PD-1 inhibitor plus 5-FU+oxaliplatin+Docetaxel+Leucovorin (FLOT) in a real-world cohort.

**Methods:**

This single-center retrospective study included patients with resectable, HER2-negative LAGC/EGJ adenocarcinoma (cT3–4b, any N+, M0; ECOG 0–1) treated between July 2020 and July 2025. Patients received neoadjuvant PD-1 inhibitor plus SOX or PD-1 inhibitor plus FLOT (3–5 cycles) followed by D2 gastrectomy. The primary endpoint was pathological complete response (pCR). Secondary endpoints included major pathological response (MPR), radiologic response (RECIST v1.1), perioperative outcomes, treatment-related adverse events (CTCAE v5.0), recurrence-free survival (RFS), and overall survival (OS). Survival was analyzed using Kaplan–Meier methods and Cox proportional hazards models.

**Results:**

Overall, 247 patients were enrolled (PD-1+SOX, n=141; PD-1+FLOT, n=106) with comparable baseline characteristics. Radiologic outcomes were similar between groups (ORR: 70.92% vs 66.98%, p=0.507; DCR: 87.23% vs 85.85%, p=0.752). Pathological responses did not differ significantly (pCR: 20.57% vs 16.98%, p=0.477; MPR: 37.59% vs 31.13%, p=0.292). Any-grade treatment-related adverse events occurred in 67.38% and 75.47% of patients, and grade ≥3 events in 19.15% and 26.42%, respectively; no treatment-related deaths occurred. R0 resection rates were high (100% vs 99.06%). Operative time and estimated blood loss were higher in the PD-1+FLOT group (p=0.010 and p=0.040), while postoperative complication rates were comparable. With median follow-up of 21 months (12–52) and 20 months (10–46), there were no significant differences in OS (HR 1.155, 95% CI 0.624–2.138) or RFS (HR 0.805, 95% CI 0.461–1.405). In multivariable analyses, non-MPR was an independent risk factor for both OS and RFS.

**Conclusions:**

Neoadjuvant PD-1 inhibitor plus SOX and plus FLOT yielded comparable response rates, survival outcomes, and safety profiles in patients with resectable LAGC/EGJ adenocarcinoma. PD-1+SOX was associated with less operative burden, and MPR remained independently associated with OS and RFS, supporting its value for risk stratification and treatment optimization.

## Introduction

Locally advanced gastric cancer (LAGC) remains a major global health burden (1), and neoadjuvant/perioperative therapy has been widely adopted to improve resectability and long-term outcomes (2–4). However, the choice of the optimal chemotherapy backbone continues to be a pivotal clinical decision, as it directly affects treatment intensity, tolerability, perioperative safety, and the likelihood of achieving meaningful pathological response. In Western practice, 5-FU+oxaliplatin+Docetaxel+Leucovorin (FLOT) is commonly used as the perioperative standard (5, 6), whereas S-1+oxaliplatin (SOX) is frequently preferred in East Asia owing to its convenience and established efficacy in Asian populations (7–9). The phase II DRAGON III trial recently provided direct evidence that neoadjuvant FLOT and SOX may yield comparable efficacy and safety, supporting both regimens as acceptable backbones in resectable LAGC (10, 11).

With accumulating evidence supporting the addition of PD-1 inhibitors to perioperative chemotherapy, neoadjuvant immunochemotherapy has rapidly entered routine clinical practice. Nevertheless, the optimal chemotherapy partner for PD-1 blockade remains undefined (12–14). Although FLOT and SOX are among the most widely used and guideline-endorsed regimens, head-to-head data comparing PD-1 inhibitor plus FLOT versus PD-1 inhibitor plus SOX remain lacking. Therefore, we conducted a real-world comparative cohort study to evaluate the efficacy and safety of neoadjuvant PD-1 inhibitor combined with SOX versus FLOT in patients with resectable LAGC, and to explore prognostic factors associated with survival.

## Methods

### Study design

This retrospective cohort study compared neoadjuvant PD-1 inhibitor plus SOX versus PD-1 inhibitor plus FLOT in patients with locally advanced gastric/gastroesophageal junction (G/EGJ) adenocarcinoma treated at the Department of Digestive Surgery of Xijing hospital between July 2020 and July 2025. The study was approved by the institutional ethics committee (KY20242419-C-1) and conducted in accordance with the Declaration of Helsinki.

Eligible patients met the following criteria: age 18–80 years; histologically confirmed HER2-negative G/EGJ adenocarcinoma; resectable locally advanced disease on pretreatment imaging (cT3–4b, any N+, M0) with ECOG performance status 0–1; receipt of neoadjuvant PD-1 inhibitor combined with SOX or FLOT with an intent for curative surgery; and subsequent D2 lymphadenectomy. Key exclusion criteria included confirmed metastatic disease, addition of PD-1 inhibitor only after chemotherapy initiation, combined anti-angiogenic therapy or radiotherapy in the primary analysis, other malignancies, and missing key exposure/outcome information.

### Treatment regimens

Planned neoadjuvant regimens were: PD-1 inhibitor + FLOT: 5-fluorouracil 2600 mg/m² (24-h infusion) + leucovorin 200 mg/m² + oxaliplatin 85 mg/m² + docetaxel 50 mg/m², every 2 weeks (3–5 cycles). PD-1 inhibitor + SOX: oxaliplatin 130 mg/m² on day 1 + S-1–80 mg/m² twice daily on days 1–14, every 3 weeks (3–5 cycles). PD-1 inhibitors included nivolumab, pembrolizumab, serplulimab, tislelizumab, and sintilimab. The patients underwent surgery within 4–6 weeks after completing neoadjuvant therapy. All patients underwent D2 radical gastrectomy in accordance with the Japanese gastric cancer treatment guidelines. According to the specific stage of the patient, corresponding chemotherapy regimens are provided for treatment. The chemotherapy regimen is the same as before.

### Endpoints and assessment

The primary endpoint was pathological complete response (pCR). Secondary endpoints included major pathological response (MPR), radiologic response, R0 resection rate, perioperative outcomes, treatment-related adverse events, recurrence-free survival (RFS), and overall survival (OS). Adverse events were graded per CTCAE v5.0, and radiologic response was assessed by RECIST v1.1 (15) (CR/PR/SD/PD; ORR=CR+PR; DCR=CR+PR+SD). Pathological response was evaluated using the Becker tumor regression grading (TRG) system: TRG 1a (0% residual tumor), TRG 1b (<10%), TRG 2 (10–50%), and TRG 3 (>50%). pCR was defined as TRG 1a, and MPR as TRG 1a/1b (16). Conversion refers to conversion from minimally invasive surgery (laparoscopic/robotic) to open surgery.

### Follow−up

After treatment, patients were followed every 3 months for the first 2 years and every 6 months from years 3 to 5 by outpatient visits and/or telephone contact. Routine assessments included physical examination, imaging studies, and laboratory tests. RFS was calculated from the date of surgery to recurrence; OS was calculated from the date of surgery to death from any cause or last follow-up. The data cutoff (last follow-up) was December 2025.

### Statistical analysis

Data analysis was performed using SPSS statistical software, version 23.0. Continuous variables are presented as the mean and standard deviation and were compared using the t-test if normally distributed, or as the median and interquartile range and were compared using the Wilcoxon rank-sum test if nonnormally distributed; categorical data are presented as numbers and percentages and were compared using the Pearson χ2 test or Fisher’s exact test, as appropriate. Survival was estimated using Kaplan–Meier curves and compared with the log-rank test; hazard ratios (HRs) with 95% confidence intervals (CIs) were derived from Cox proportional hazards models. All statistical tests were two-sided. A p-value <0.05 was considered statistically significant.

## Results

### Patient characteristics

**Figure 1:**
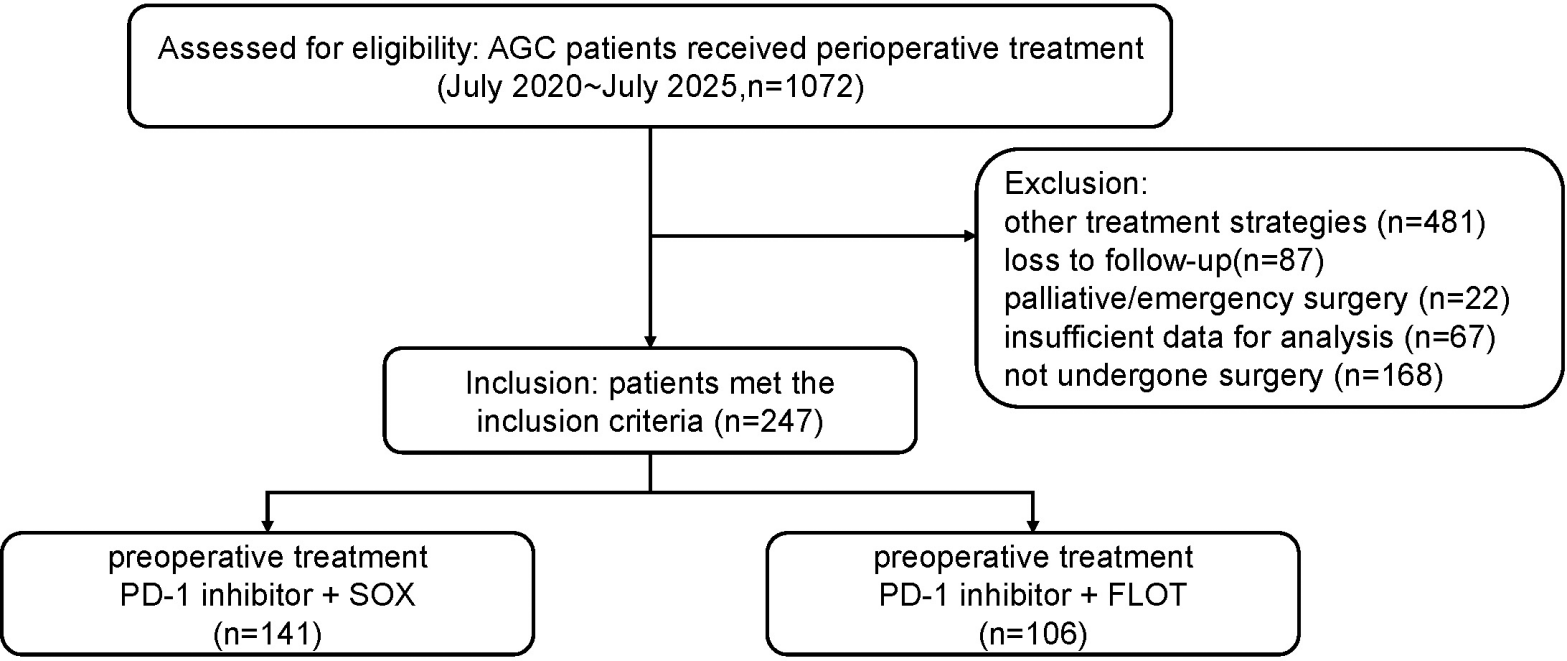
Flow diagram of the patient selection process.

After applying the eligibility criteria, 247 patients were enrolled, including 141 treated with PD-1 inhibitors + SOX and 106 treated with PD-1 inhibitors + FLOT. The process of patient selection is presented in **Figure 1**. Baseline variables were comparable between the two groups, including age (≥60 years: 57.45% vs 58.21%), sex, BMI category, ECOG score, primary site, differentiation grade, and clinical T stage distribution. PD-L1 CPS was unavailable in 40.43% (PD-1 inhibitors + SOX) and 33.02% (PD-1 inhibitors + FLOT), while MMR status was unavailable in 13.48% and 17.92%, respectively. baseline characteristics are summarized in **Table 1**. PD-1 inhibitors and cycles were similar between the two groups (**Supplementary Figure 1**), and there was no significant difference in postoperative adjuvant chemotherapy (**Supplementary Table 1**).

**Table 1:** Baseline characteristics of patients.

Characteristic	PD-1 inhibitors + SOX	PD-1 inhibitors + FLOT	P value
	n=141	n=106	
Age (Years)			0.869
≥60	81 (57.45)	62 (58.49)	
<60	60 (42.55)	44 (41.51)	
Sex			0.121
Male	107 (75.89)	89 (83.96)	
Female	34 (24.11)	17 (16.04)	
BMI (Kg/m^2)			0.533
≥25	34 (24.11)	22 (20.75)	
<25	107 (75.89)	84 (79.25)	
ECOG score			0.943
0	103 (73.05)	77 (72.64)	
1	38 (26.95)	29 (27.36)	
Primary site			0.545
Esophago-gastric junction	69 (48.94)	56 (52.83)	
Gastric	72 (51.06)	50 (47.17)	
Degree of differentiation			0.902
Low	88 (62.41)	61 (61.32)	
Medium	42 (29.79)	31 (29.25)	
High	11 (7.80)	10 (9.43)	
Clinical T-stage, n (%)			0.313
cT3	68 (48.23)	58 (54.72)	
cT4	73 (51.77)	48 (45.28)	
Clinical N-stage, n (%)			0.410
cN0	18 (12.77)	20 (18.87)	
cN1	65 (46.10)	39 (36.79)	
cN2	33 (23.40)	26 (24.53)	
cN3	25 (17.73)	21 (19.81)	
CPS			
<1	23 (16.31)	25 (23.58)	0.293
≥1	61 (43.26)	46 (43.40)	
<5	55 (39.00)	47 (44.34)	0.925
≥5	29 (20.57)	24 (22.64)	
<10	70 (49.64)	62 (58.49)	0.486
≥10	14 (9.93)	9 (8.49)	
Unknown	57 (40.43)	35 (33.02)	
Mismatch Repair (MMR)			
pMMR	111 (78.72)	81 (76.42)	0.581
dMMR	11 (7.80)	6 (5.66)	
Unknown	19 (13.48)	19 (17.92)	

BMI, Body Mass Index; ECOG, Eastern Cooperative Oncology Group; CPS, Combined Positive Score.

### Tumor regression

Tumor response was assessed in all patients. Radiological response assessment by RECIST v1.1 showed that CR, PR, SD, and PD were achieved in 21 (14.89%), 79 (56.03%), 23 (14.80%), and 18 (16.31%) patients in the PD-1 inhibitors+SOX group, and 16 (15.09%), 55 (51.89%), 20 (18.87%), and 15 (14.15%) patients in the PD-1 inhibitors+FLOT group, respectively. The ORR was 70.92% vs 66.98% (p=0.507), and the DCR was 87.23% vs 85.85% (p=0.752). After surgery, pathological regression was evaluated using Becker TRG. The pCR rate was 20.57% vs 16.98% (p=0.477), and the MPR rate was 37.59% vs 31.13% (p=0.292). As detailed in **Table 2**.

**Table 2:** Efficacy of PD-1 inhibitors+SOX group and PD-1 inhibitors+FLOT group.

Category, No. (%)	PD-1 inhibitors + SOX	PD-1 inhibitors + FLOT	P value
	n=141	n=106	
Radiological Assessment			0.917
CR	21 (14.89)	16 (15.09)	
PR	79 (56.03)	55 (51.89)	
SD	23 (14.80)	20 (18.87)	
PD	18 (16.31)	15 (14.15)	
ORR	100 (70.92)	71 (66.98)	0.507
DCR	123 (87.23)	91 (85.85)	0.752
Pathological Assessment			0.489
TRG 1a	29 (20.57)	18 (16.98)	
TRG 1b	24 (17.02)	15 (14.15)	
TRG 2	56 (39.71)	40 (37.74)	
TRG 3	32 (22.70)	33 (31.13)	
pCR	29 (20.57)	18 (16.98)	0.477
MPR	53 (37.59)	33 (31.13)	0.292
Nerve invasion			0.260
Yes	75 (53.19)	64 (60.38)	
No	66 (46.81)	42 (39.62)	
Vessel invasion			0.306
Yes	56 (39.72)	49 (46.23)	
No	85 (60.28)	57 (53.77)	
Pathology T-stage, n (%)			0.972
ypT0	30 (21.28)	19 (17.92)	
ypT1	23 (16.31)	18 (16.98)	
ypT2	10 (7.09)	8 (7.55)	
ypT3	48 (34.04)	39 (36.79)	
ypT4	30 (21.28)	22 (20.75)	
Pathology N-stage, n (%)			0.961
ypN0	76 (53.90)	59 (55.66)	
ypN1	22 (15.60)	14 (13.21)	
ypN2	19 (13.48)	15 (14.15)	
ypN3	24 (17.02)	18 (16.98)	
Pathology TNM stage, n (%)			0.881
ypT0N0	29 (20.57)	18 (16.98)	
Stage I	31 (21.99)	23 (21.70)	
Stage II	34 (24.11)	29 (27.36)	
Stage III	47 (33.33)	36 (33.96)	

CR, Complete Response; PR, Partial Response; SD, Stable Disease; PD, Progressive Disease; ORR, Objective Response Rate; DCR, Disease Control Rate; TRG, Tumor Regression Grade; MPR, Major Pathological Response.

### Safety

During neoadjuvant treatment, any-grade treatment-related adverse events occurred in 95 (67.38%) patients in the PD-1 inhibitors+SOX group and 80 (75.47%) in the PD-1 inhibitors+FLOT group; grade ≥3 events occurred in 27 (19.15%) and 28 (26.42%), respectively. The most common hematological toxicities were leukopenia, thrombocytopenia, and neutropenia, while the most common non-hematological toxicities included ALT/AST increased. No treatment-related deaths were observed (**Table 3**).

**Table 3:** Safety of PD-1 inhibitors+SOX group and PD-1 inhibitors+FLOT group.

Events No. (%)	PD-1 inhibitors+SOX (n=141)		PD-1 inhibitors+FLOT (n=106)		P value^a^	P value^b^
	All grades	≥Grade 3	All grades	≥Grade 3		
Treatment-related AEs						
Any	95 (67.38)	27 (19.15)	80 (75.47)	28 (26.42)	0.166	0.174
ALT/AST increased	57 (40.43)	10 (7.09)	47 (44.34)	11 (10.38)	0.537	0.36
Leukopenia	47 (33.33)	9 (6.38)	39 (36.79)	9 (8.49)	0.572	0.528
Thrombocytopenia	32 (22.70)	13 (9.22)	32 (30.19)	11 (10.38)	0.183	0.761
Neutropenia	30 (21.27)	8 (5.67)	26 (24.53)	5 (4.72)	0.546	0.739
Anaemia	26 (18.44)	8 (5.67)	22 (20.75)	8 (7.55)	0.649	0.554
Vomit	17 (12.06)	1 (0.71)	12 (11.32)	0 (0)	0.859	0.385
Nausea	14 (9.93)	1 (0.71)	10 (9.43)	0 (0)	0.897	0.385
Decreased appetite	11 (7.80)	0 (0)	8 (7.55)	0 (0)	0.941	NA
Abdominal pain	5 (3.55)	0 (0)	3 (2.83)	0 (0)	0.753	NA
Hand foot syndrome	2 (1.42)	0 (0)	0 (0)	0 (0)	0.218	NA
Peripheral neuropathy	2 (1.42)	0 (0)	5 (4.72)	0 (0)	0.122	NA

AE, Adverse Event; ALT, Alanine aminotransferase; AST, Aspartate aminotransferase. a: p value for all grades AEs, b: p value for ≥Grade 3 AEs.

### Surgical and postoperative outcomes

Radical gastrectomy with D2 lymphadenectomy was performed as planned. R0 resection was achieved in 141 (100%) patients in the PD-1 inhibitors+SOX group and 105 (99.06%) in the PD-1 inhibitors+FLOT group. The conversion rate, retrieved lymph nodes, type of surgery, surgical approach, resection extent, and postoperative length of stay were comparable between the two groups, however, the operation time and estimated blood loss of the PD-1 inhibitors+FLOT group were significantly higher than those of the PD-1 inhibitors+SOX group (p=0.010 and 0.040). Overall postoperative complications occurred in 11 (7.80%) and 9 (8.49%) patients in the PD-1 inhibitors+SOX and PD-1 inhibitors+FLOT groups, respectively (p=0.844). Grade III–V complications were observed in 7 (4.96%) vs 7 (6.60%) patients. Postoperative mortality was 0.71% vs 0% (p=0.385) (**Table 4**).

**Table 4:** Surgical and postoperative outcomes of PD-1 inhibitors+SOX group and PD-1 inhibitors+FLOT group.

Variable	PD-1 inhibitors+SOX	PD-1 inhibitors+FLOT	P value
	n=141	n=106	
Surgical outcomes			
Total operation time, min (median, IQR)	235 (82.5)	265 (96.25)	***0.010***
Estimated blood loss, ml (median, IQR)	50 (50)	100 (150)	***0.040***
Conversion (n, %)	2 (1.42)	1 (0.94)	0.736
Length of incision, cm (median, IQR)	8 (5)	8 (5)	0.825
Distal margin, cm (median, IQR)	6 (6.5)	8 (6.4)	0.065
Proximal margin, cm (median, IQR)	2.5 (3)	2 (1.53)	0.174
Number of retrieved LNs (mean ± SD)	24.95 ± 7.77	26.31 ± 7.62	0.171
R0 (n, %)	141 (100)	105 (99.06)	0.248
Type of surgery			0.494
Laparoscopic surgery	126	92	
Robotic surgery	8	10	
open surgery	7	4	
Surgical approach			0.188
Transabdominal approach	123	86	
Thoracoabdominal approach	18	20	
Resection extent			0.502
Subtotal gastrectomy	38	25	
Total gastrectomy	85	62	
Proximal gastrectomy with/without esophagectomy	18	19	
Postoperative outcomes			
Postoperative hospital stay,day (median, IQR)	6 (2.5)	6 (4)	0.167
**Overall postoperative complications (n,%)**	11 (7.80)	9 (8.49)	0.844
Clavien-Dindo classification (n,%)			
Grades I-II	4 (2.84)	2 (1.89)	
Grade I	0 (0)	1 (0.94)	
Grade II	4 (2.84)	1 (0.94)	
Grades III-V	7 (4.96)	7 (6.60)	
Grade III	5 (3.55)	4 (3.77)	
Grade IV	1 (0.71)	3 (2.83)	
Grade V	1 (0.71)	0 (0)	
Details (n,%)			
Pleural effusion/abscess	3 (2.13)	4 (3.77)	
Anastomosis leakage	6 (4.26)	4 (3.77)	
Pulmonary	2 (1.42)	3 (2.83)	
Respiratory failure	2 (1.42)	3 (2.83)	
Intra-abdominal abscess	2 (1.42)	2 (1.89)	
Bacteremia	1 (0.71)	0 (0)	
Multiple organ failure	1 (0.71)	0 (0)	
Mortality (n,%)	1 (0.71)	0 (0)	0.385

IQR, interquartile range; LN, lymph nodes.The bold and italicized text indicates statistically significant differences.

### Survival

**Figure 2:**
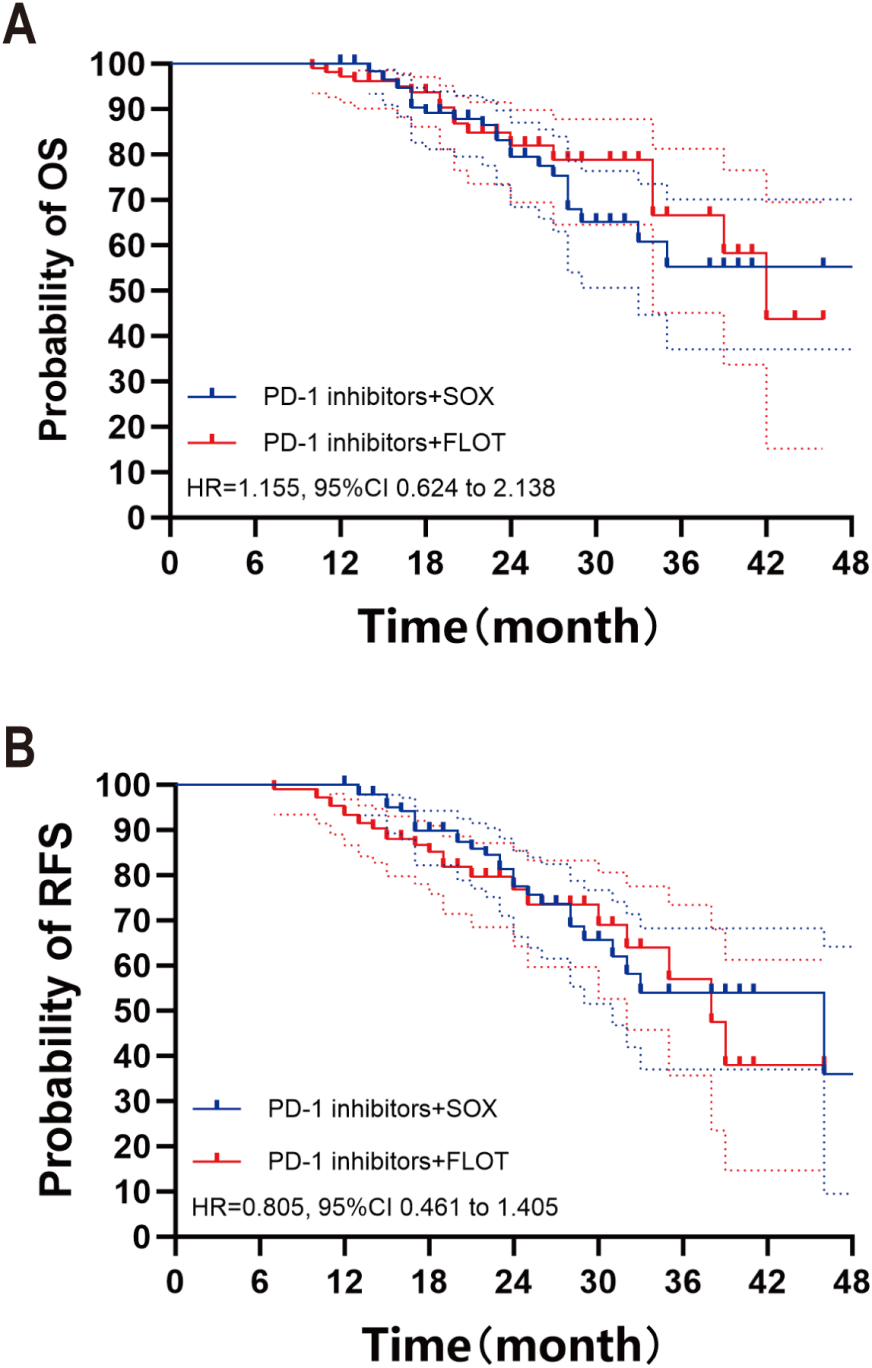
Overall survival **(A)** and recurrence-free survival **(B)** of patients in two groups.

With the data cutoff, the median follow-up was 21 months (range, 12–52), 28 patients recurrence and 25 patients died in the PD-1 inhibitor + SOX group, 20 months (range, 10–46), 24 patients recurrence and 17 patients died in the PD-1 inhibitor + FLOT group. The median OS was not reached in the PD-1 inhibitor + SOX group and was 42 months in the PD-1 inhibitor + FLOT group; the median RFS was 46 months and 38 months, respectively. Kaplan–Meier curves for OS and RFS are shown in **Figure 2**. No significant differences were observed between groups, with an OS hazard ratio (HR) of 1.155 (95% CI 0.624–2.138) and an RFS HR of 0.805 (95% CI 0.461–1.405) for PD-1 inhibitor + SOX versus PD-1 inhibitor + FLOT.

We further performed Cox regression analyses for OS and RFS including eight variables (age, ECOG performance status, tumor location, histological differentiation, treatment regimen, MPR, lymphovascular invasion, and perineural invasion). Non-MPR remained an independent risk factor for both OS and RFS (**Supplementary Tables 2**, **3**).

## Discussion

This single-center, real-world retrospective cohort study compared the efficacy and safety of neoadjuvant PD-1 inhibitor plus SOX versus PD-1 inhibitor plus FLOT in patients with locally advanced G/EGJ adenocarcinoma. Our results demonstrated that PD-1 inhibitor combined with either FLOT or SOX achieved comparable long-term outcomes (OS and RFS), pathological responses (pCR and MPR), and overall safety profiles. Additionally, PD-1 inhibitor plus SOX was associated with shorter operative time and lower intraoperative blood loss compared with PD-1 inhibitor plus FLOT.

Substantial geographic differences have long existed in the selection of perioperative chemotherapy backbones: FLOT is more commonly adopted (5, 6) in Western practice and has also been reported in China (17), whereas SOX is widely used across East Asia (9, 18). The DRAGON III trial directly compared FLOT and SOX and suggested broadly similar efficacy and safety in the neoadjuvant chemotherapy setting (10, 11). With immunotherapy emerging as a major perioperative strategy, numerous studies on perioperative immunotherapy combined with chemotherapy for gastric cancer have been conducted. The DANTE (IKF-s633) and NEOSUMMIT-01 trails have shown that immunotherapy combined with chemotherapy can significantly increase the pathological response rate (12, 14). The KEYNOTE-585 and MATTERHORN trials further demonstrated the benefits of immunochemotherapy on survival outcomes (19, 20), direct evidence remains limited regarding which chemotherapy backbone is optimal for combination with PD-1 blockade. In routine clinical practice, our comparative evaluation demonstrated similar ORR and DCR between the two regimens, with no significant differences in pCR or MPR. Survival analyses further indicated no statistically significant differences in OS and RFS, suggesting that, in this cohort, the overall clinical benefit achieved by combining PD-1 inhibitors with either backbone was comparable. From a real-world perspective, these findings support the notion that both SOX and FLOT can serve as feasible “platform” regimens for perioperative immunochemotherapy in East Asian populations, and regimen selection may be individualized based on tolerability, treatment convenience, and institutional perioperative management experience. Notably, recent phase III trials have reinforced the role of perioperative immunochemotherapy: KEYNOTE-585 demonstrated improvement in event-free survival (EFS) (19), and MATTERHORN reported that adding immunotherapy to a FLOT backbone further significantly improved EFS and OS (20). In this context, our prognostic analyses are clinically informative. Cox regression showed that MPR was independently associated with OS and RFS, underscoring pathological response as a meaningful indicator of true tumor sensitivity to systemic therapy and a potential determinant of long-term outcomes beyond the choice of chemotherapy backbone (21). This interpretation is consistent with the long-term follow-up of DRAGON III, which suggests that the impact of pathological response on survival may outweigh that of regimen selection per se. Therefore, in the era of immunochemotherapy, optimizing perioperative strategies may rely less on switching chemotherapy backbones and more on increasing the likelihood of achieving MPR, for example through more precise patient selection, strengthened perioperative management, and early strategy modification for non-responders. At present, many studies focus on multi-omics data to predict the survival of patients with tumor (22–24), and to accurately manage the treatment strategy of patients, so as to achieve survival benefits. Among them, the prediction model of efficacy response to immunotherapy is the most widely studied. With the progress of research, it can bring more accurate guidance for clinical treatment (25–27).

With respect to safety, the overall incidence of treatment-related adverse events was similar between groups. Although grade ≥3 events were numerically more frequent in the PD-1+FLOT group, the difference was not statistically significant, and no treatment-related deaths occurred. Importantly, operative time and intraoperative blood loss were significantly higher in the PD-1+FLOT group than in the PD-1+SOX group. This may be related to the greater intensity of the triplet regimen, potentially leading to more pronounced myelosuppression and inflammatory responses, increased tissue edema, or fibrosis, thereby complicating surgical exposure and dissection. For frail patients or those with higher perioperative risk, SOX as the chemotherapy backbone in combination with PD-1 blockade may confer an advantage in terms of surgical burden by potentially reducing surgical complexity while maintaining comparable oncological outcomes; however, prospective studies are warranted to confirm causality and elucidate underlying mechanisms.

Several limitations should be acknowledged. First, as a retrospective, single-center study, our analysis is subject to selection bias and residual confounding, and the PD-1 inhibitors used were not fully uniform between groups, which may introduce drug-specific effects. Second, missing data for CPS and MMR limited the robustness of biomarker-based stratification. Third, although no significant differences in OS/RFS were observed, the maturity of follow-up and the number of events may have limited statistical power; larger prospective studies with longer follow-up are needed for confirmation.

In conclusion, in this real-world cohort, neoadjuvant PD-1 inhibitor plus SOX and PD-1 inhibitor plus FLOT demonstrated comparable pathological response, perioperative safety, and OS/RFS, supporting both regimens as reasonable chemotherapy backbones for perioperative immunochemotherapy. PD-1+SOX showed potential advantages in operative time and intraoperative blood loss. More importantly, the independent association of MPR with OS and RFS suggests that pathological response may serve as a key anchor for clinical decision-making and risk stratification. Future prospective studies with standardized biomarker testing are required to define the optimal immunochemotherapy backbone and the patient subgroups most likely to benefit.

## Data Availability

The original contributions presented in the study are included in the article/**Supplementary Material**. Further inquiries can be directed to the corresponding author.
