# Supplementary material for: Neoadjuvant PD-1 inhibitor combined with FLOT versus SOX for locally advanced gastric cancer: a retrospective cohort study
Source: Front Immunol. 2026 Feb 25;17:1782029. doi: 10.3389/fimmu.2026.1782029 (PMC12975886; doi:10.3389/fimmu.2026.1782029)

Supplementary Material

**sTable 1.** Postoperative adjuvant therapy details

**sTable 2.** Univariate and multivariate analysis of overall survival

**sTable 3.** Univariate and multivariate analysis of recurrence-free survival

**sFigure 1.** PD-1 inhibitors details for two groups

**sTable1.** Postoperative adjuvant therapy details

| **Item** | **PD-1 inhibitors+SOX (n=141)** | **PD-1 inhibitors+FLOT (n=106)** | **p value** |
| --- | --- | --- | --- |
| **Time to start adjuvant therapy after surgery (weeks)** | 5 (4-8) | 5 (4-9) | 0.019 |
| **Patients with adjuvant chemotherapy** | 117 | 86 | 0.707 |
| **Cycles** | 4 (2-5) | 4 (2-5) | 0.395 |
| **patients with adjuvant PD-1 inhibitor** | 73 | 49 | 0.388 |
| **patients completed 8 treatments** | 72 | 57 | 0.673 |

**sTable2.** Univariate and multivariate analysis of overall survival

| Variables | Univariate | | Multivariate | |
| --- | --- | --- | --- | --- |
|  | HR (95% CI) | P value | HR (95% CI) | P value |
| **Age (Years)** |  |  |  |  |
| <60 | Ref. |  |  |  |
| ≥60 | 1.354(0.720-2.545) | 0.347 |  |  |
| **ECOG score** |  |  |  |  |
| 0 | Ref. |  |  |  |
| 1 | 1.348(0.716-2.538) | 0.355 |  |  |
| **Primary site** |  |  |  |  |
| Esophago-gastric junction | Ref. |  |  |  |
| Gastric | 0.673(0.362-1.251) | 0.211 |  |  |
| **Degree of differentiation** |  |  |  |  |
| Low | Ref. |  |  |  |
| Medium | 1.180(0.620-2.245) | 0.614 |  |  |
| High | 0.570(0.134-2.429) | 0.448 |  |  |
| **Therapeutic regimen** |  |  |  |  |
| PD-1 inhibitor+FLOT | Ref. |  |  |  |
| PD-1 inhibitor+SOX | 1.155(0.624-2.138) | 0.649 |  |  |
| **MPR** |  |  |  |  |
| Yes | Ref. |  |  |  |
| No | 6.492(2.314-18.214) | ***<0.001*** | 3.436(1.062-11.124) | ***0.039*** |
| **Vessel invasion** |  |  |  |  |
| Yes | Ref. |  |  |  |
| No | 0.324(0.171-0.613) | ***0.001*** | 0.742(0.361-1.526) | 0.418 |
| **Nerve invasion** |  |  |  |  |
| Yes | Ref. |  |  |  |
| No | 0.206(0.091-0.466) | ***<0.001*** | 0.454(0.169-1.223) | 0.118 |
| ECOG: Eastern Cooperative Oncology Group‌, MPR: Major Pathological Response; Italicized letters represent significant differences. | | | | |

**sTable3.** Univariate and multivariate analysis of recurrence-free survival

| Variables | Univariate | | Multivariate | |
| --- | --- | --- | --- | --- |
|  | HR (95% CI) | P value | HR (95% CI) | P value |
| **Age (Years)** |  |  |  |  |
| <60 | Ref. |  |  |  |
| ≥60 | 1.456(0.826-2.568) | 0.194 |  |  |
| **ECOG score** |  |  |  |  |
| 0 | Ref. |  |  |  |
| 1 | 1.144(0.634-2.064) | 0.656 |  |  |
| **Primary site** |  |  |  |  |
| Esophago-gastric junction | Ref. |  |  |  |
| Gastric | 0.681(0.392-1.182) | 0.172 |  |  |
| **Degree of differentiation** |  |  |  |  |
| Low | Ref. |  |  |  |
| Medium | 0.814(0.449-1.476) | 0.498 |  |  |
| High | 0.358(0.086-1.494) | 0.159 |  |  |
| **Therapeutic regimen** |  |  |  |  |
| PD-1 inhibitor+FLOT | Ref. |  |  |  |
| PD-1 inhibitor+SOX | 0.805(0.461-1.405) | 0.449 |  |  |
| **MPR** |  |  |  |  |
| Yes | Ref. |  |  |  |
| No | 7.639(2.751-21.215) | ***<0.001*** | 4.772(1.470-15.492) | ***0.009*** |
| **Vessel invasion** |  |  |  |  |
| Yes | Ref. |  |  |  |
| No | 0.329(0.185-0.584) | ***<0.001*** | 0.723(0.368-1.421) | 0.347 |
| **Nerve invasion** |  |  |  |  |
| Yes | Ref. |  |  |  |
| No | 0.242(0.118-0.497) | ***<0.001*** | 0.665(0.267-1.655) | 0.380 |
| ECOG: Eastern Cooperative Oncology Group‌, MPR: Major Pathological Response ; Italicized letters represent significant differences. | | | | |

**sFigure 1.** PD-1 inhibitors details for two groups
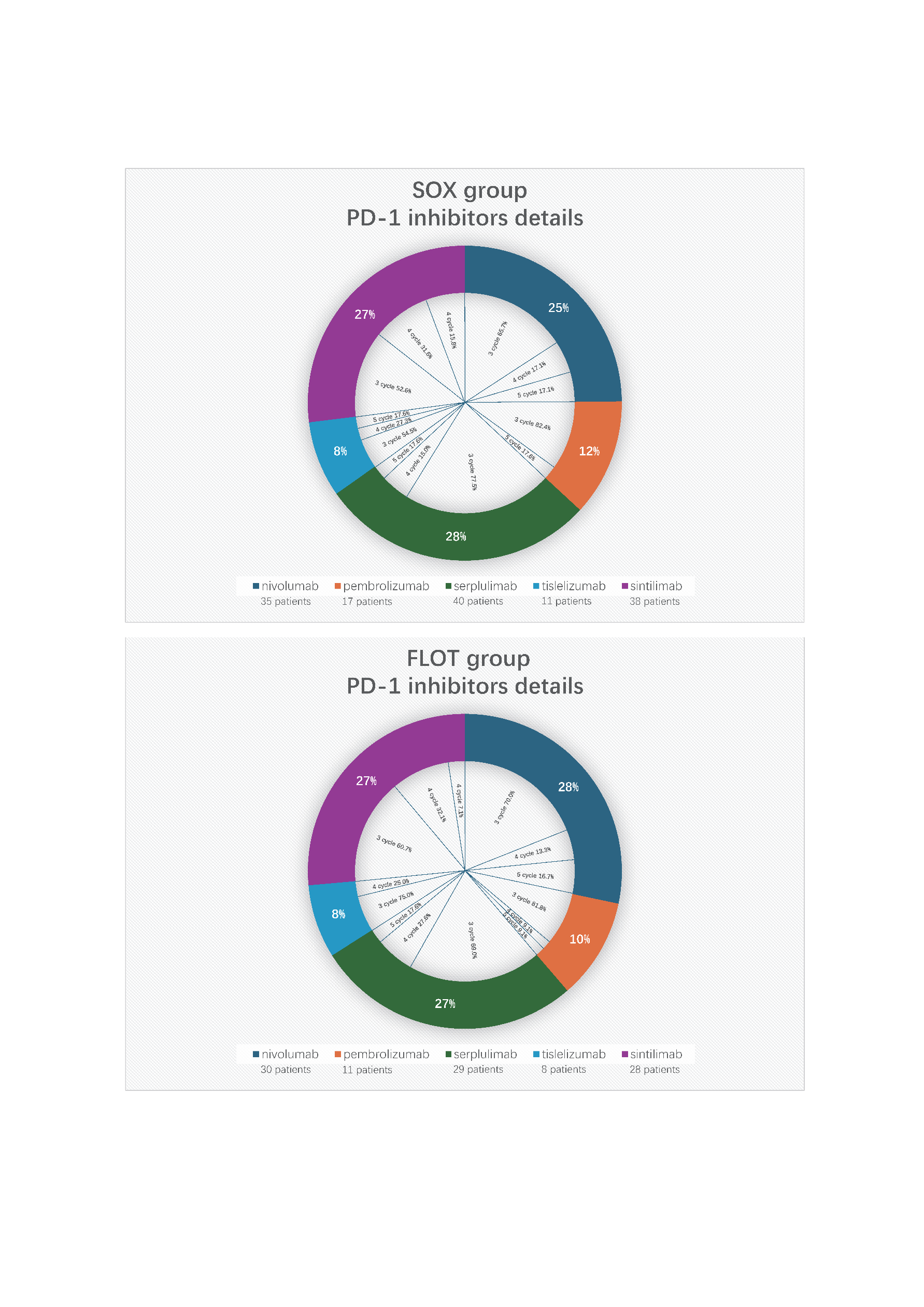

Supplement: Supplementary file 1 [file DataSheet1.docx]
